# Grey literature citations in top nursing journals: a bibliometric study

**DOI:** 10.5195/jmla.2020.760

**Published:** 2020-04-01

**Authors:** Stephen Woods, Kathleen Phillips, Andrew Dudash

**Affiliations:** University Libraries, Penn State University, University Park, PA, sjw31@psu.edu; Nursing and Allied Health Liaison Librarian, Life Sciences Library, University Libraries, Penn State University, University Park, PA, kec5013@psu.edu; University Libraries, Penn State University, University Park, PA, amd846@psu.edu

## Abstract

**Objective:**

As access to information grows in tandem with the growth of the Internet, access to grey literature also increases. Because little is known about the use of grey literature in nursing journals, the authors investigated the prevalence and types of grey literature citations in top nursing journals.

**Methods:**

We analyzed all citations (n=52,116) from articles published in 2011 in 6 top nursing journals selected from the Medical Library Association’s Nursing and Allied Health Resource Section’s 2012 “Selected List of Nursing Journals.” Grey literature citations were identified and categorized by type.

**Results:**

Grey literature accounted for 10.4% of citations across all 6 journals. Publications from governments (54.3%) and corporate organizations (26.8%) were the most common types of grey literature.

**Conclusion:**

The substantial citation of grey literature in nursing journals shows that nursing scholars seek and use this category of information. These findings have implications for teaching and learning among nursing researchers and the information professionals who serve the nursing research community.

## INTRODUCTION

Grey literature is often discussed as being problematic or described “negatively or by what it is not” [1], eliciting the perception that it is difficult to find, frequently misunderstood, or simply not used during the research process. Over time, however, the definition of grey literature has changed from its roots in the literature of reports to the currently accepted Luxembourg definition of “that which is produced on all levels of government, academics, business and industry in print and electronic formats, but which is not controlled by commercial publishers” [2], which was updated at the 2004 Sixth International Conference on Grey Literature in New York to include “where publishing is not the primary activity of the producing body” [3]. Grey literature has also evolved from Gibb and Phillips’ suggestion that it is “dimly perceived” [4], when considering the need for grey literature repositories in Europe, to Banks’ assertion that there will be an “eventual collapse of the distinction between grey and non-grey literature” [5], in the context of institutional repositories and the burgeoning open access movement. Today, the use of grey literature is flourishing, and its “distribution…in a multitude of mediums…has become widespread” [6].

Information about the citation of grey literature in nursing journals has the potential to educate researchers, practitioners, and information professionals in the allied health fields. While previous studies have examined the use of grey literature across various disciplines, there remains a gap in the discussion about the use of grey literature in scholarly nursing communication. Thus, studying the prevalence and types of grey literature citations in nursing journals can provide insight into the information used by nursing scholars and enhance information professionals’ support for the scholarly community that seeks and uses this information.

Previous bibliographic studies of nursing literature provide a bridge between research and practice [7] and have focused on utilization of research [8], origin of nursing theory [9], and avenues of communication between research and clinical literature [10], but they do not provide granular detail about the types of citations commonly found in peer-reviewed journals. In a bibliometric study, Oermann et al. provided an updated look at the use of grey literature in nursing scholarly communication and found that nearly 10% of citations in clinical and research nursing journals were to grey literature [11]. The next step to further develop the body of research on the use of grey literature in nursing journals, therefore, is to delve into the granular details of this specific subset of citations.

The existing discussion of grey literature in nursing publications is sparse and provides little clarity or direction for researchers, practitioners, or information professionals who are seeking to broaden their grey literature knowledgebase. In 2006, *Nursing Times* published an overview of grey literature, providing nurses with a guide to defining, searching for, and citing grey literature [12]. That same year, the Medical Library Association’s (MLA’s) Nursing and Allied Health Resource Section (NAHRS) collaborative Task Force on Mapping the Nursing Literature mapped the literature of general nursing and sixteen specialties of nursing [13].

While these mapping studies provided valuable insight into nursing research and publishing trends, they did not discuss, or even categorize, grey literature. Although the NAHRS study identified grey literature citations, they were only categorized as “Internet,” “government documents,” or “miscellaneous.” In a follow-up of the NAHRS study in 2016, Watwood performed a bibliographic analysis of pediatric nursing literature and used the same categories of “Internet,” “government documents,” or “miscellaneous” [14].

Today, however, grey literature encompasses much more than Internet, government documents, and miscellaneous sources, and this study takes the next step of identifying the prevalence and, more specifically, definition of the types of grey literature cited in nursing journals. As established by Pelzer and Wiese in their study of veterinary journals, the bibliometric analysis of grey literature citations in scholarly publications can provide key insights into research and publishing trends [15]. As a modification of Pelzer and Wiese’s study, the objective of the present study was to investigate the prevalence and type of grey literature cited in top nursing journals.

## METHODS

The authors employed citation analysis to determine the incidence of grey literature appearing in the bibliographies of articles published in six top nursing research journals.

NAHRS mapped the nursing literature to identify core journals in 2006 and created a “Selected List of Nursing Journals” in 2012 [16]. Differing from Pelzer and Weise’s approach to creating their own list of core veterinary journals, we used the 2012 updated evidence-based list of nursing and interdisciplinary journal titles as our selection bank because this list serves as a key tool for collection development and publication opportunities for librarians and nurses. Titles were ranked by the number of research articles published and by research percentage from data provided in the 2012 NAHRS “Selected List of Nursing Journals” [17]. We created a combined rank from these totals and selected the six titles with the smallest totals (Table 1). These titles were selected because they represented a sample that provided a balance between practical and research as well as domestic and international scholarly communication. The final titles by ranking were:

1. Journal of Clinical Nursing
2. Infection Control and Hospital Epidemiology
3. Patient Education and Counseling
4. Journal of Advanced Nursing
5. Scandinavian Journal of Caring Sciences
6. Maternal and Child Health Journal

**Table 1:** Ranking of 2012 Nursing and Allied Health Resource Section (NAHRS) list of nursing journals

Nursing and Allied Health Resource Section (NAHRS) journals 2012	# Research articles rank	Research % rank	Combined rank
*Journal of Clinical Nursing*	1	1	2
*Patient Education and Counseling*	4	5	9
*Infection Control and Hospital Epidemiology*	5	4	9
*Journal of Advanced Nursing*	3	13	16
*Maternal and Child Health Journal*	11	6	17
*Scandinavian Journal of Caring Sciences*	14	3	17

*International Journal of Nursing Studies*	7	14	21
*Qualitative Health Research*	9	17	26
*Cancer Nursing*	20	9	29
*Journal of Women’s Health*	6	26	32
*Midwifery*	17	19	36
*Journal of Nursing Management*	13	27	40
*Nursing Research*	28	12	40
*International Journal of Nursing Practice*	23	18	41
*Journal of School Health*	18	24	42
*Health Care for Women International*	27	16	43
*Nurse Education Today*	12	36	48
*Journal of Pain and Symptom Management*	10	40	50
*American Journal of Public Health*	2	49	51

**Figure 1:**
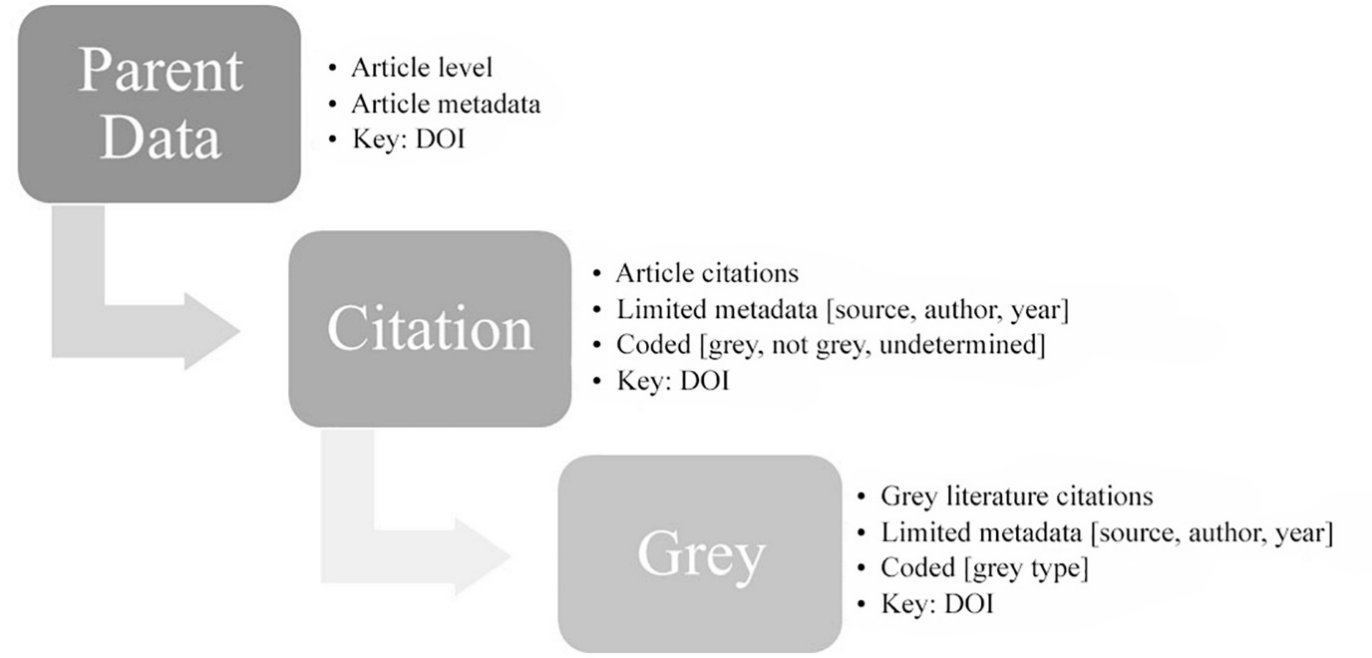
Flowchart of data extraction and coding process

Using this 2012 list of core journals, we analyzed citation data from articles published in 2011 to determine the prevalence of grey literature appearing in the top six journals. The complete metadata for articles published in 2011 was pulled from Web of Science to create a parent article data set for each journal (Figure 1). Only citations from publications tagged by Web of Science as editorials, articles, and reviews were included; correspondence, letters, retractions, corrections, proceedings, and biographies were excluded. The list of articles from Web of Science was compared to those on the official journals’ websites. Missing articles were added to the parent article data set with information from the journal website to match the metadata from articles pulled from Web of Science.

A separate citation data set was created for each journal. For articles pulled from Web of Science, these data were initially wrangled from information found in a text file in the citation reference field of the parent articles. From this text file, we created a record for each citation that contained at least an author and source. Some citation records, mostly those from serial publications, also included year, volume, page, and digital object identifier (DOI). We added the corresponding DOI for each citation as a key from the article in the parent article dataset. We checked the total number of citations for each article pulled from Web of Science against the number of citations shown on the journal website, if available. If the numbers did not match, the data were checked for the missing or additional citations. Missing Web of Science citations were added to the citation data set to match the metadata schema.

Citations that we identified as published in common serial titles were coded as not grey literature, whereas those with a source or author that we easily determined to be a government entity or corporate organization were coded as grey literature. Citations in need of further analysis were marked as undetermined. Following this initial pass, citations that were marked as grey or undetermined were more closely examined. If the article’s citation did not provide sufficient information to allow its categorization, we analyzed the full text of the cited publication, when available, or obtained additional information about the publication from other databases. Citations categorized as grey literature were further coded as one of the following categories: (1) conference proceeding, (2) government, (3) news, (4) corporate organization, (5) thesis or dissertation, or (6) higher education. Publications cited as “submitted for publication” were verified as published and coded accordingly. Different from Pelzer and Wiese’s study [15], we created separate categories for higher education and news and used a broader definition of news to eliminate “miscellaneous” as a category. Coding was performed by a library specialist in policy and government resources to provide consistency in interpreting all citations needing further analysis.

Higher education citations included any type of source from academic institutions that were not published by a commercial publisher, including correspondence, curricula, departmental publications, and unpublished research. University press publications were not included. Corporate organizations included nonprofit organizations, research and policy entities, and corporate publications that were not distributed through commercial publishers. News included newspapers, newsletters, and other forms of media communication, such as blogs and social media. Conference proceedings included publications that were distributed through professional societies and associations or through commercial publishers; only those commercially published proceedings sponsored by higher education or a corporate organization were included in this category. Government citations encompassed reports from intergovernmental agencies (e.g., World Health Organization, Organisation for Economic Co-operation and Development) as well as foreign, federal, state, and local governments. Joint corporate organization and higher education publications were assigned to the sponsoring organization. Theses and dissertations were not included in higher education but were coded as a separate category.

The percentage of total grey literature citations and the percentage breakdown of specific types of grey literature were calculated for each journal title.

## RESULTS

Six top research journals valuable to the field of nursing in 2011 were selected for citation analysis. A total number of 1,467 articles published in these journals yielded a total of 52,116 citations. Grey literature comprised 5,399 of these citations (10.4%), with a range of 6.7% to 16.8% of citations across journals. On average, there were 3.7 grey literature citations per article (Table 2).

**Table 2:** Grey literature citations in nursing journals in 2011

	# Articles	# Citations	# of grey literature citations	# of grey literature citations per article (average)	% of grey literature citations per journal
*MCHJ*	174	6,411	1,075	6.2	16.8%
*ICHE*	191	4,507	531	2.8	11.8%
*JCN*	406	14,314	1,555	3.8	10.9%
*JAN*	258	10,770	1,058	4.1	9.8%
*SJCS*	101	3,861	359	3.6	9.3%
*PEC*	337	12,253	821	2.4	6.7%
Total	1,467	52,116	5,399	3.7	10.4%

MCHJ: Maternal and Child Health Journal; ICHE: Infection Control and Hospital Epidemiology; JCN: Journal of Clinical Nursing; JAN: Journal of Advanced Nursing; SJCS: Scandinavian Journal of Caring Sciences; PEC: Patient Education and Counseling.

**Figure 2:**
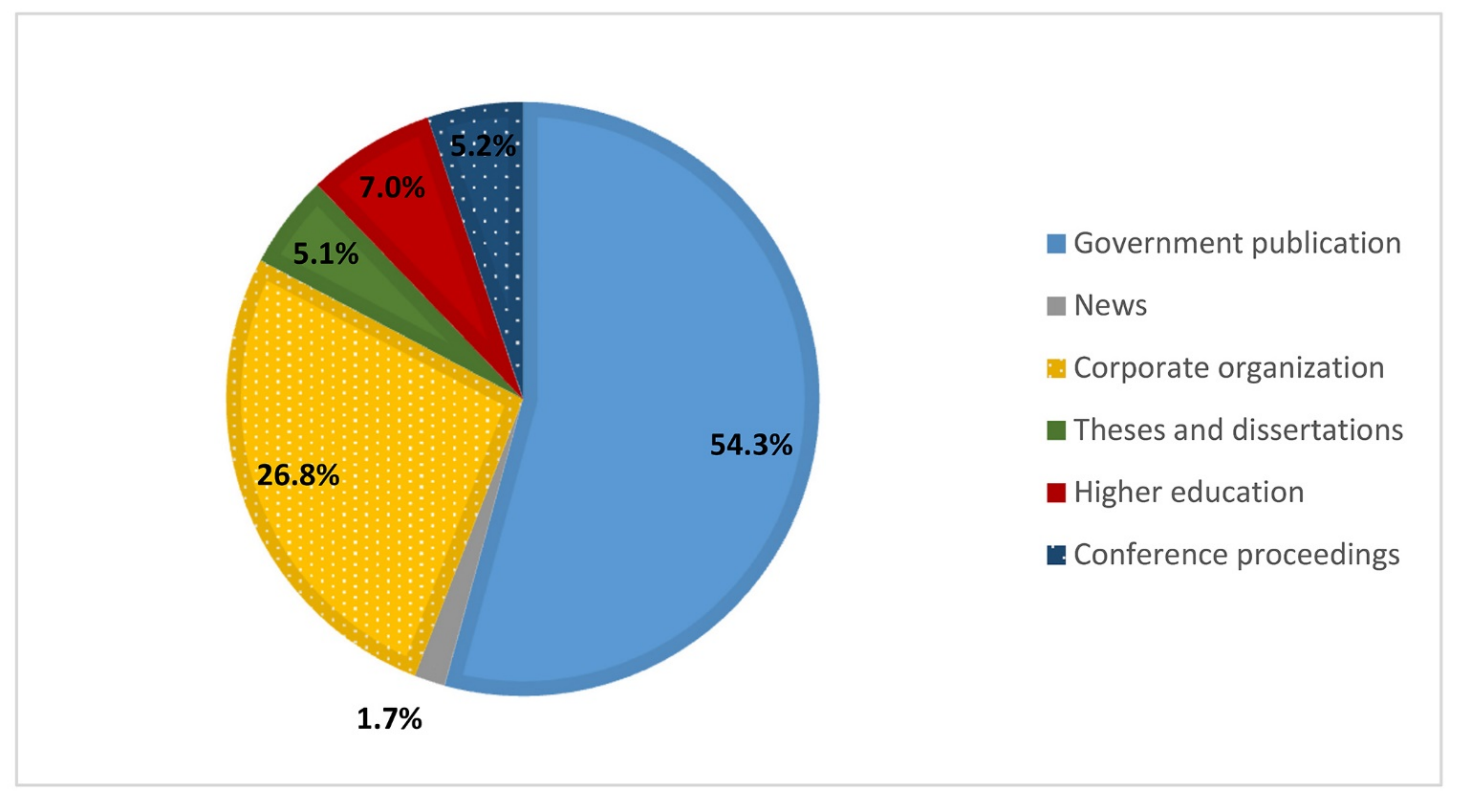
Percentage of grey literature types across journals

**Figure 3:**
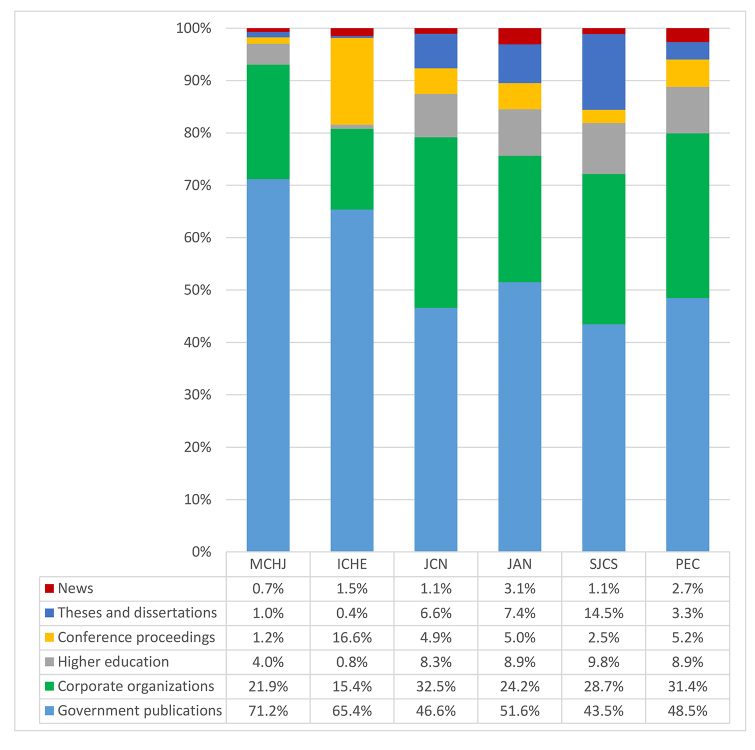
Grey literature type and prevalence for each journal

Figure 2 shows an overall breakdown of grey literature citations by type, and Figure 3 reports the prevalence of each type for each journal. Across all 6 journals, government publications accounted for over half of the grey literature cited and appeared most frequently in the *Maternal Child Health Journal*. Corporate organization publications accounted for 26.8% of grey literature cited and appeared most frequently in the *Journal of Clinical Nursing*. Higher education publications represented 7.0% of the total grey literature citations and appeared most frequently in the *Scandinavian Journal of Caring Sciences* and *Patient Education and Counseling*. Conference proceedings citations, at 5.2%, were almost as common as higher education publication citations and appeared most frequently in *Infection Control and Hospital Epidemiology.* Theses and dissertations accounted for 5.1% of grey literature citations and were cited most frequently in the *Scandinavian Journal of Caring Sciences.* Only 1.7% of grey literature citations were to news sources.

## DISCUSSION

This analysis was born out of the curiosity to investigate the prevalence of grey literature citations in key nursing journals to inform researchers’, practitioners’, and information professionals’ perceptions of grey literature and related teaching and practice. Investigating the specific types of grey literature citations broadens understanding of how grey literature is used in nursing scholarly communication.

The relatively high prevalence of citations to grey literature (10.4%) in this study indicated its significant use in a key selection of nursing journals and was consistent with other findings [11]. Rather than using a sample of articles, we comprehensively evaluated a large number of journal articles (n=52,116) to reduce sampling error. We found that grey literature was consistently cited across all 6 selected journals (range, 6.7%–16.8% of citations), indicating that nursing researchers were finding and using grey literature in their publications. In addition, the average of almost 4 grey literature citations per journal article reinforced that grey literature was a common and recognized source of valuable information. The variety of types of grey literature identified in this study spoke to the range of information that authors seek to support their research objectives and findings.

Government publications were the most frequently cited type of grey literature, emphasizing their importance and the continual need to use and assess government information. Publications from corporate organizations were the second most frequently cited type of grey literature, demonstrating the value of corporate information in the academic research environment. We speculate that these two types of grey literature will remain predominant and sought after in the future.

While conference proceedings did not play a prevalent role in our study, there is evidence that this venue has the potential to be a vital component of scholarly communication [18], with Ania proposing that “proceedings…provide the medium for reporting [grey literature]” [19]. Pelzer and Wiese’s bibliometric study of veterinary literature reported that 50.1% of grey literature citations fell in the conference category [15], which was much higher than the frequency of conference proceedings citations in peer-reviewed nursing journals that was found in our study. Based on the results of Rowe’s recent mapping study of poster presentation citations [18], however, the frequency with which conference proceedings are cited in nursing journals may grow over time.

Interestingly, we found that news was the least cited type of grey literature in nursing journals. Although many scholars use blogs as a platform to integrate personal experiences with scholarly research and news outlets can act as a public point of access to and understanding of scholarly research, these sources did not appear to have a strong presence in scholarly nursing communication.

A limitation of this research was that grey literature types were coded by only one member of the research team, although guidelines were discussed and debated by all team members to reach a consensus of understanding and achieve consistency in the coding process. Also, we recognized that using Web of Science to identify and assign publication types might have produced some discrepancies. While the final list of selected journals for this study were international in scope, they were chosen from the NAHRS list and, thus, had an English language bias, which might have influenced our results.

Pelzer and Wiese predicted that “the shifting of information resources to the Internet is likely to reduce the incidence of grey literature in veterinary medicine even further,” but our results contradicted this statement. If anything, the evolving information ecosystem grants easier and more extensive access to grey literature sources, prompting greater use of different types of grey literature in scholarly communication. Pelzer and Wiese also anticipated the appearance of a new type of grey literature in the form of “community email forums and specialty discussion groups” [15]. While these were not identified as categories in this study, our introduction of higher education publications as a new type of grey literature and omission of a “miscellaneous” category spoke to an identifiable shift in where researchers search for and how they use grey literature.

Librarians and information professionals have the opportunity to promote discussions about the credibility of and potential for bias in grey literature as access to information expands. The prevalence and types of grey literature cited in nursing journals indicate its steady incorporation into scholarly communication, and the implications of these findings can inform teaching and learning among nursing researchers and information professionals who serve the nursing research community. Grey literature makes a substantial contribution to nursing research, and information professionals can apply this knowledge to their instruction, research, and practice.

## 

**Stephen Woods**, sjw31@psu.edu, University Libraries, Penn State University, University Park, PA

**Kathleen Phillips**, kec5013@psu.edu, Nursing and Allied Health Liaison Librarian, Life Sciences Library, University Libraries, Penn State University, University Park, PA

**Andrew Dudash**, amd846@psu.edu, University Libraries, Penn State University, University Park, PA
